# Effectiveness of adapted self-help plus (SH+) to reduce psychological distress among university students in Indonesia (APRESIASI): protocol of a randomized controlled trial

**DOI:** 10.1186/s40359-025-03026-y

**Published:** 2025-07-08

**Authors:** Dhini Andriani, Fredrick D. Purba, Anke B. Witteveen, Neily Zakiyah, Marit Sijbrandij

**Affiliations:** 1 Department of Clinical, Neuro- and Developmental Psychology, Vrije Universiteit Amsterdam, Amsterdam, Netherlands; 2 Department of Psychology, Faculty of Psychology, Universitas Padjadjaran, Jatinangor, Indonesia; 3 World Health Organization (WHO) Collaborating Centre, Vrije Universiteit Amsterdam, Amsterdam, Netherlands; 4 Amsterdam Public Health, Mental Health, Amsterdam, Netherlands; 5 Department of Pharmacology and Clinical Pharmacy, Faculty of Pharmacy, Universitas Padjadjaran, Jatinangor, Indonesia; 6 Center of Excellence in Higher Education for Pharmaceutical Care Innovation, Universitas Padjadjaran, Bandung, Indonesia

**Keywords:** Self-help plus (SH+), University students, Indonesia, Psychological distress

## Abstract

**Background:**

University students in Indonesia are exposed to stressors such as high academic task load, financial strains, and relationship problems. Therefore, they are at risk of developing symptoms of common mental disorders, such as depression and anxiety (i.e., psychological distress). However, there is a gap between the number of mental health professionals and the number of students in need of a psychological intervention. Self-Help Plus (SH+), a guided group-based stress management intervention developed by the WHO, was adapted to the population and context to address barriers in accessing mental health care among students in Indonesia. The aim of the APRESIASI study is to assess the effectiveness and cost-effectiveness of SH + in reducing psychological distress among university students in Indonesia.

**Methods:**

The SH + intervention will be tested in a pragmatic superior randomized controlled trial (RCT) with two parallel arms. SH + will be tested among Indonesian university students with symptoms of psychological distress (Patient Health Questionnaire-9; PHQ-9 ≥ 5.5 < 20). The participants (*n* = 296) will be randomized into either the intervention group that receives SH + with enhanced care as usual (ECAU), or into the ECAU only with a 1:1 allocation ratio. The primary outcome is the reduction in psychological distress assessed with the Patient Health Questionnaire Anxiety and Depression Scale (PHQ-ADS) at three-month follow-up. The secondary outcomes are symptoms of depression, anxiety, perceived stress, functioning, resilience, quality of life, identified problems, treatment acceptability, and cost-effectiveness.

**Discussion:**

To our knowledge, SH + is the first a transdiagnostic, face-to-face, and group-based intervention to be tested for its effectiveness in reducing psychological distress in Indonesia. If shown to be effective, SH + has the potential to be scaled up across university settings.

**Trial registration:**

ISRCTN15761598, 14/07/2023.

**Supplementary Information:**

The online version contains supplementary material available at 10.1186/s40359-025-03026-y.

## Background

Emerging adulthood reflects the period during which individuals aged 18 to 29 transitions from adolescence into adulthood. During this developmental period, they are faced with living away from their parents, pursuing education, entering the workforce, and finding a partner [1]. However, this period is also marked by a high prevalence of common mental disorders such as depression and anxiety [2, 3]. A study of emerging adults in Indonesia reported a 28.86% prevalence of depression [4] and most emerging adults in a qualitative study reported feeling anxious, with half of the respondents reporting self-harm and having suicidal thoughts [5].

The period of emerging adulthood coincides with the period of going to university. Compared with the general population, psychological distress appears to be more common and severe among university students [6]. The WHO Mental Health Survey International College students project revealed that more than 30% of students at 19 universities in eight countries screened positive for at least one common mental health disorder [7]. In the context of Indonesia, previous studies have indicated that university students exhibit symptoms of psychological distress [8], with high level of stress ranging from 37 to 50% [9, 10], a prevalence of depression between 18.8% and 25% [10, 11], and an anxiety prevalence of 51% [10]. Another study found that Indonesian university students had an increased risk of self-harm and suicide attempt [12].

Given the high prevalence of mental health problems among university students, there is a growing concern in universities to address the mental health problems of their students. Therefore, the establishment of counselling centres for students is part of a government initiative called SIMKATMAWA (System of Performance Information and Student Governance) [13]. While these centres may provide important support and reflect the growing awareness of mental health issues in higher education, there are currently no factual data on how many universities in Indonesia have mental health facilities, the qualifications of the mental health professionals involved, or the number of students utilizing these services. Furthermore, as an alternative, students can visit mental health professionals outside universities. However, there is a large gap between the number of people who need mental health interventions and the availability of such services, known as the mental health gap [14]. In the university context, there is a gap between the number of undergraduates, which exceeds eight million [15], and the availability of clinical psychologists, which is only 4000 [16]. Therefore, the implementation of low-intensity [17] and transdiagnostic interventions is proposed to meet the needs of emerging adults with common and multiple mental health problems [18, 19].

The World Health Organization (WHO) developed a range of scalable low-intensity psychological interventions that are based on transdiagnostic and task-sharing principles. One of these interventions is Self Help Plus (SH+), which is a group-based (up to 30 people) stress management course for adults, delivered by a non-specialist. It was developed based on acceptance commitment therapy (ACT) [20]. SH + consists of a pre-recorded program delivered in a group setting over five two-hour sessions, supplemented by a self-help book named *Doing What Matters in Times of Stress: An illustrated Guide* [21].

SH + has been adapted and implemented in humanitarian settings in Uganda [22], Turkey [23], refugees resettling in European countries [24], and workers in nursing facilities [25]. Furthermore, based on three RCTs in those sites, SH + was found to be effective in reducing psychological distress in adults and preventing the onset of mental disorders (e.g., depression), as were improvements in general health functioning, and subjective well-being [22, 24, 25] and in reducing economic and societal costs related to disability [26].

In the present study, we have adapted SH + to the Indonesian language and culture. We will conduct a randomized controlled trial (RCT) to compare the effectiveness of SH + combined with enhanced care as usual (ECAU) to ECAU only in reducing psychological distress in university students in Indonesia.

## Goals and hypotheses

The primary objective of the study is to evaluate the effectiveness and cost-effectiveness of a culturally adapted SH + intervention combined with ECAU among university students in Indonesia at a three-month follow-up (main time point) in reducing psychological distress. The secondary objectives are to assess depression and anxiety symptoms separately, as well as perceived stress, functioning, quality of life, resilience, identified problems, feasibility, and acceptability. In addition, moderator variables influencing the reduction in psychological distress, anxiety, and depression symptoms and improvements in functioning and quality of life will be explored.

Previous research on SH + among South Sudanese female refugees in Uganda found that SH + reduced psychological distress three months after the intervention [22]. In addition, a follow-up period of three months is long enough to observe the sustained effects of the intervention while minimizing loss to follow-up and recall bias. In the context of our university student population, a three-month timeframe also aligns with academic terms, thereby reducing the risk of attrition due to changes in academic circumstances or different types of activities. For example, some students may have to enrol in an internship programme or live in a rural community for a full semester. Therefore, we expect that SH + with ECAU (SH+/ECAU) will be superior to the ECAU only in reducing psychological distress (primary outcome) at three-month follow-up, as well as the secondary outcomes of depression and anxiety, perceived stress, improved functioning, quality of life, resilience, and identified problems. Furthermore, we expect that SH+/ ECAU will report lower healthcare costs than ECAU-only.

## Method

### Research design

**Fig. 1:**
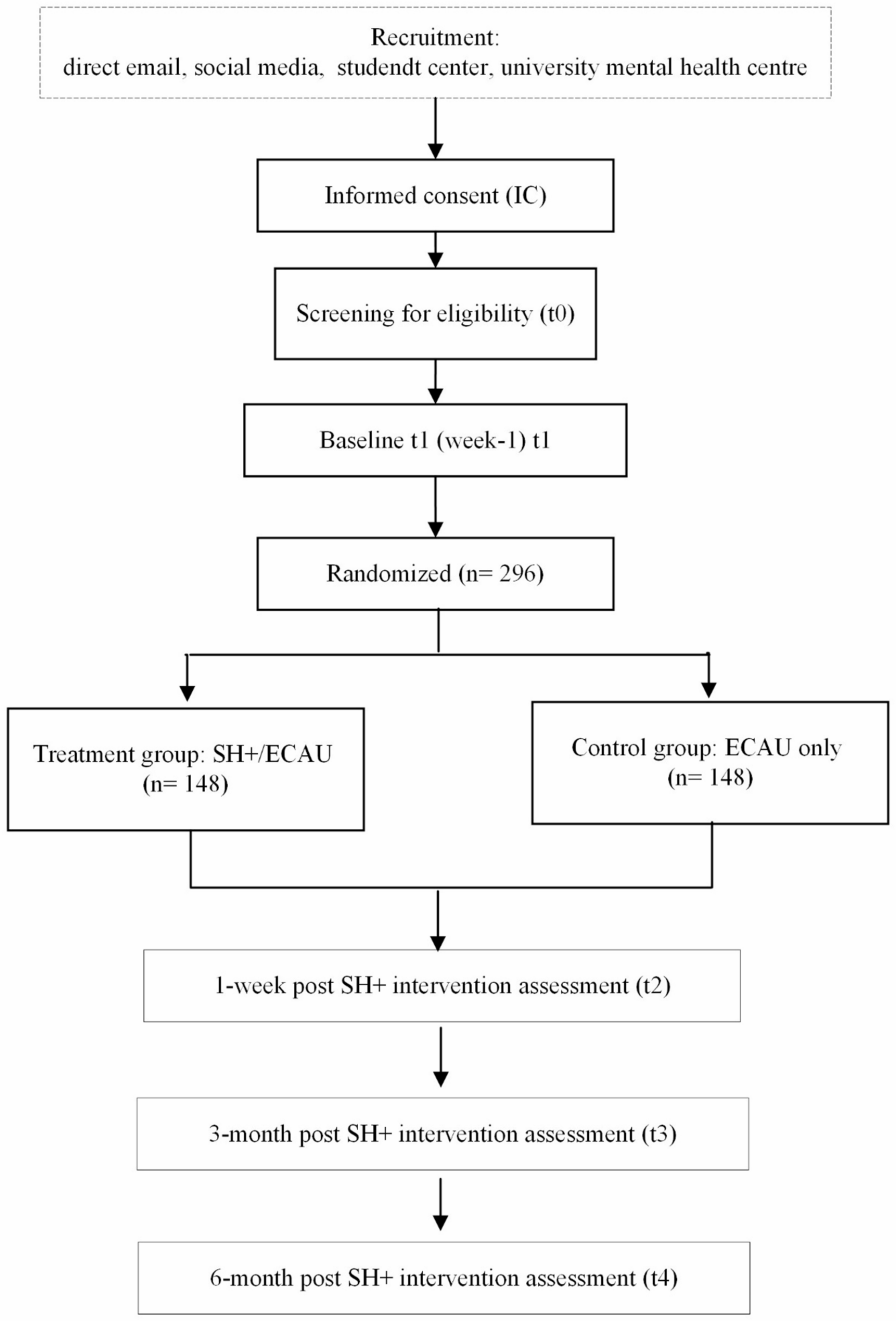
Flowchart of the randomized controlled trial according to CONSORT

This is a pragmatic, single-blind RCT with two parallel arms. This RCT will be conducted following the Consolidated Standard of Reporting Trials (CONSORT) [27] (see Fig. 1).

### Study setting

The Adapted Self-Help Plus (SH+) to Reduce Psychological Distress among University Students In Indonesia (APRESIASI) project is led by Vrije Universiteit Amsterdam (VU) in collaboration with Universitas Padjadjaran (Unpad), Indonesia. Participants will be recruited from two universities, namely Unpad and Institut Teknologi Bandung (ITB), located near the city of Bandung, West Java.

### Participants

#### Inclusion criteria

- University students aged between 17 and 29 years old.
- Score ≥ 5.5 [28] on the Patient Health Questionnaire-9 (PHQ-9) [29].
- Willingness to attend five sessions of SH+.

#### Exclusion criteria

- Acute medical conditions requiring immediate hospitalization;
- score ≥ 20 (severe depression symptoms) on PHQ-9 based on Kroenke et al. (2001);
- indication of imminent risk of suicide or self-harm or other life-threatening risk based on Suicide Ideation Scale (SIS) [30] with score ≥ 21.89 [31];
- evidence of severe cognitive impairment related to mental, neurological or substance abuse based on the PM + observation checklist [32];
- started, stopped or significantly changed pharmacotherapy in the previous eight weeks;
- specialized psychological treatment (e.g., cognitive behavioural therapy, psychoanalysis) initiated or discontinued in the past eight weeks.

### Procedure

#### Recruitment

Several strategies will be used to recruit participants for this study. As a first strategy to identify participants, an online mental health survey will be conducted among university students. Advertisements about mental health surveys will be posted on universities’ and students’ social media. This advertisement will contain information about the mental health survey and the link for participating in the survey. In the survey link, the students will read the information about the study and are asked to provide consent before they fill out the survey form. Participants with psychological distress (PHQ-9 ≥ 5.5), who are eligible according to the above-mentioned inclusion and exclusion criteria and who give permission to be followed-up after completion of the survey, will be contacted for further interviews. During this interview, the RCT will be explained, and they will be asked whether they are interested in participating in the RCT.

As a second strategy, advertisements about the RCT will be distributed through all social media at the universities, faculties, work units and student organisations. This advertisement will include a short explanation of the study, the contact details of the research assistant and a form to take part in the study. If anyone has questions about the study or needs further information about participation, they can contact the research assistant via e-mail or WhatsApp. In addition, the university counselling centre will be asked to actively promote RCT to students through their regular activities.

If a student expresses interest in participating in the study, they will fill in the registration form, which consists of their name, email address, contact details, and confirmation of their availability to attend the scheduled SH + sessions. The research assistant then sends the information letter of the project, the digital personal informed consent link, and privacy statement to their email. They will have one week to read the information and decide whether to participate. The research assistant will send out a reminder on the 6th and 10th days after the information letter has been sent out.

SH + is a group-based intervention, so each participating university will need to enrol at least 40–60 participants. The SH + session will start after one and a half months of recruitment. We will continue to keep the recruitment process open until the required number of participants has been reached.

#### Screening

Screening (t_0_) will be carried out via a Zoom video call (20–30 min) with a trained research assistant and consists of a self-report questionnaire (sociodemographic information and PHQ-9). If the result of PHQ-9 ≥ 5.5 but < 20, the participant will be screened for exclusion criteria by asking specific questions (e.g., “are you currently receiving psychological counselling or psychotherapy?”) and by an observation checklist completed by the research assistant (e.g., “does the participant understand you?”). Screening will stop if the participant meets any of the exclusion criteria.

#### Assessment

Data will be collected through self-report assessments at four times points: *t*_*1*_ (baseline – sent at least 10 days before the SH + to the participants already enrolled), *t*_*2*_ (post-SH+, week-7), *t*_*3*_ (main time point – 3 months after SH+), and *t*_*4*_ (follow-up – 6 months after SH+) with the Castor Electronic Data Capture (EDC) software program [33]. Each assessment will be provided via e-mail with a link to the self-report questionnaire to each participant and can be completed on any device with internet access. The time to complete each assessment is approximately 20–30 min. If an assessment remains incomplete, the participant will receive a maximum of three reminders on days 3, 7, and 10 through various communication channels to encourage retention. The participants will receive IDR 50,000 e-money for each completed assessment, with a maximum of four e-money awards (*t*_*1*_ to *t*_*4*_).

#### Assessors

The screening process is carried out by a trained research assistant with at least a bachelor’s degree in psychology who currently receives training to become a psychologist. During a video call, the research assistant will explain the study once again. The research assistant will be blinded to the group allocation.

#### Randomization

The participants will be randomly assigned to either the intervention or the control group at a 1:1 allocation ratio, using randomization with four and six blocks. The randomization will be performed by CASTOR EDC. Due to the nature of the study, the participant will know their group allocation, either it is an SH+/ ECAU or ECAU only. The research coordinator will know the results of the randomization.

#### Trial status

As of December 2024, the RCT has not yet been initiated. The first inclusion is expected in March 2025.

#### Process evaluation

Following the RCT, participants who consent to follow-up research will be approached to participate in the process evaluation. A qualitative process evaluation will be conducted to assess the satisfaction and acceptability of SH + and to identify the barriers and support to engagement in and adherence to the intervention. In addition, this evaluation aims to explore opportunities for scaling up the implementation of the SH + program for university students within the mental health system available at the university.

### Study measures

Below (Table 1) is the schedule of enrolment, intervention, and assessment of the RCT which followed the Standard Protocol Items: Recommendations for Intervention Trials (SPIRIT) [34].

**Table 1:** Schedule of enrolment, interventions, and assessments for RCT of SH + within according to SPIRIT

	STUDY PERIOD						
	Enrolment	Screening	Baseline	Randomization	Post-intervention	Follow-up 1 (main endpoint nt)	Follow-up 2
		t_0_	Week 1 (t_1_)		Week 7 (t_2_)	3 months (t_3_)	6 months (t_4_)
**Enrolment**:							
Informed consent	X						
Eligibility screening		X					
Allocation				X			
**Intervention**:							
SH+ & ECAU				↔			
ECAU				↔			
**Assessments**:							
Sociodemographic		X	X		X	X	X
PHQ-9			X		X	X	X
GAD-7			X		X	X	X
PSS			X		X	X	X
WHODAS 2.0			X		X	X	X
EQ-5D-5L			X		X	X	X
MIMIS			X		X	X	X
PSYCHLOPS			X		X	X	X
CSRI			X		X	X	X
Process evaluation (conducted after *t*_3)_						X	

ECAU: enhanced care as usual; PHQ-9: Patient Health Questionnaire-9; GAD-7: General Anxiety Disorder-7; PSS: Perceived Stress Scale; WHODAS 2.0: WHO Disability Assessment Schedule 2.0; EQ-5D-5L: EuroQol 5 dimensions, 5 levels; PSYCHLOPS: Psychological Outcomes Profiles; MIMIS: Mainz Inventory of Micro-stressors; CSRI: Client Service Receipt Inventory

#### Screening measures

The Patient Health Questionnaire-9 (PHQ-9) is used as a screening tool. The PHQ-9 is a self-administered questionnaire consisting of nine items that can be scored from 0 (not at all) to 3 (almost every day) [29]. PHQ-9 score range from 0 to 27 with 0–4 indicating no depressive symptoms, 5–9 mild, 10–14 moderate, 15–19 moderately severe, and 20–27 severe. The PHQ-9 has been validated for Bahasa Indonesia with a Cronbach’s alpha of 0.885 and a cut-off score of 5.5, indicating that people have experienced depression [28].

Indication of imminent risk of suicide or self-harm or other life-threatening risk will be assessed using Suicide Ideation Scale (SIS) which is a 10-item self-report questionnaire to identify ideation that distinguishes covert suicidal ideation from more overt or intense ideation [30]. The SIS scored from 1 (never or none of the time) to 5 (always or a great many times) based on how the participant has felt or behaved in the past week, with a total score range from 10 to 50. The SIS has been validated to Bahasa Indonesia with a Cronbach’s alpha 0.92 and cut-off score ≥ 21.89 [31].

Cognitive impairment will be assessed using an observation checklist from the PM + manual [32].

#### Primary outcome measure

The primary outcome and end point will be the reduction in psychological distress as measured by the total score on the Patient Health Questionnaire Anxiety and Depression Scale (PHQ-ADS) [35, 36] at three-month follow-up. The PHQ-ADS is the validated combined sum score of the Patient Health Questionnaire (PHQ-9) [29] and the Generalized Anxiety Disorder (GAD-7) questionnaires [37]. Psychological distress, as measured by the PHQ-ADS, will also be collected at one-week and six-month follow-up.

#### Secondary outcome measure

All secondary measurements will be collected at one week, three months, and six months post-treatment. Depression symptoms are measured using the PHQ-9 [29]. The PHQ-9 is a self-administered questionnaire consisting of nine items that can be scored from 0 (not at all) to 3 (almost every day), with a total score ranging from 0 to 27. The PHQ-9 has been validated in Bahasa Indonesia demonstrating good internal consistency (Cronbach’s alpha of 0.885), and a cut-off score of 5.5, which indicates that people have symptoms of depression [38].

Anxiety symptoms are measured using the GAD-7 [37]. It consists of seven items with response options ranging from 0 (not at all) to 3 (almost every day), with a total score ranging from 0 to 21. The GAD-7 was translated into Bahasa Indonesia and validated with a Cronbach’s alpha 0.867 and a cut-off score of 6 [39].

The Perceived Stress Scale (PSS) will be used to measure perceived stress. The PSS is a measure of an individual’s perception of recent stress [40]. The items are designed to measure how unpredictable, uncontrollable and overwhelming respondents feel their lives are. The questions in the PSS ask about feelings and thoughts over the past month. The PSS consists of 10 items that are scored from 0 to 4 with the response options being ‘*never*’, ‘*almost never*’, ‘*sometimes*’, ‘*fairly often*’ and ‘*very often*’. The PSS in Bahasa Indonesia showed very good item reliability (0.98) and person reliability (0.79) [41].

General functioning will be measured by the WHO Disability Assessment Schedule 2.0 (WHODAS 2.0) -12 items [42]. The WHODAS 2.0 is a generic assessment tool that can be used to measure health and disability at the population level or in clinical practice. It consists of 12 items that assess six activity domains: understanding and communicating, getting around, self-care, getting along with people, life activities and participation in society. For each item, the participants indicate the level of difficulty (none, mild, moderate, severe, extreme). The Bahasa Indonesia version of WHODAS 2.0–12 items showed good internal consistency (Cronbach’s alpha between 0.634 and 0.853) and test-retest reliability (0.764 and 0.866) [43].

Improvement in Quality of life will be measured by the EQ-5D-5L. The EQ-5D-5L instrument, provided by the EuroQol Group, consists of two sections. The first section includes five items covering five health state dimensions: mobility, self-care, usual activities, pain/discomfort, and anxiety/depression. Each dimension has five response levels: no problems, slight problems, moderate problems, severe problems, and extreme problems/unable to. These responses can be combined into a 5-digit health state profile; for example, ‘11122’ indicates no problems in mobility, self-care, and usual activities, with slight problems in pain/discomfort and anxiety/depression [44]. The second section of the EQ-5D-5L is the visual analogue scale (VAS), where respondents rate their overall health status on a vertical scale from 0 (‘worst imaginable health’) to 100 (‘best imaginable health’). The recall period for both sections of the EQ-5D-5L is ‘today.’ The EQ-5D-5L has been widely used and is available in over 150 languages, including Bahasa Indonesia, and has good test-retest reliability (Gwet’s agreement coefficient: 0.85–0.99 and percentage agreement: 90–99%) [45]. Indonesian’s utility [46] weights are applied to the EQ-5D-5L data, and changes in participants’ quality of life years gained between the intervention and control groups are determined.

We assess resilience, which is defined as the ability to maintain good mental health despite adversity [47]. This involves linking changes in mental health to exposure to stressors. The Mainz Inventory of Micro-stressors (MIMIS), which was recently developed to measure objective micro-stressors of modern life in the last 14 days [48], will be used for this purpose [49]. Each MIMIS item has two response scales: a four-point Likert scale indicating the frequency with which a stressor occurred, ranging from 0 (did not happen/ almost never) to 3 (almost every day), and a five-point Likert scale indicating the extent to which the stressor caused mental strain, ranging from 0 (this situation did not happen) to 4 (severe impact).

The Psychological Outcome Profiles instrument (PSYCHLOPS) assesses self-identified problems [50]. The PSYCHLOPS consists of four questions that cover three domains: problems (two questions), and functioning (one question), and wellbeing (one question). The participants are asked to give free answers to the problem and the functional domain. Responses are scored on a six-point scale, with producing a maximum score of 20. The pre- and post-therapy versions of PSYCHLOPS have the same four questions with additional in the post-therapy version about overall evaluation questions, a self-rated outcome ranging from ‘much better’ to ‘much worse’.

The Client Service Receipt Inventory (CSRI) [51] will be used to examine cost-effectiveness and health service utilization over the eight months (t1, t2, t3, t4). The questionnaire consists of questions concerning what kind of health services (public, private, university, or others), number of visits, type (in-person/ online), duration of contact, travel and waiting time, and travel cost. Additionally, trial costs will be collected (e.g. training, book printing, supervision).

#### Other measures

Sociodemographic information will be collected during screening and at all-time points. Data will be collected on demographics (e.g., gender, age, religion), education (e.g., GPA, year of study, field of study), and living conditions (e.g., housing, living allowance). Contamination questions will be asked at the one-week post-test and the three-month follow-up in both arms. For the intervention group, participants will be asked, ‘Did you discuss SH + with others?’ For the control group, participants will be asked, ‘Did you know about SH+?’ if the answer is ‘yes’, there will be several follow-up questions (e.g., ‘How did you know about SH+’, ‘Did you learn about the SH + such as reading the book or listening to the audio?’).

*Translation and adaptation of the study measures*. PSYCHLOPS, MIMIS, and CSRI were not yet available in Bahasa Indonesia. Therefore, in this project, we adapted these three instruments. In general, the adaptation process included translation, synthesis, back-translation, and final synthesis. The Indonesian research team (DA and FDP) carried out the synthesis part, while hired professionals’ translators did the translation into Bahasa Indonesia and back-translation into English (different translator).

In the adaptation of PSYCHLOPS, there were no changes between the English version and the Bahasa Indonesia version. For the adaptation of MIMIS, we used the result of a qualitative interview with university students (*N* = 30) in Indonesia about their problems. The results of these interviews informed new items related to daily challenges reported by university students in Indonesia, resulting in a version of the MIMIS adapted for Indonesian university students. We adapted the CSRI based on the health system in Indonesia and had a consultation with an expert in health economics.

### Intervention

#### Self-Help plus (SH+)

The participants in the experimental arm will receive SH + and ECAU (see the ECAU section below). SH + is a five-session, group-based (20–30 people) stress management intervention for adults [20]. The five sessions last 90–100 min each and take place once a week for a total of five weeks. SH + consists of a pre-recorded audio course and an illustrated self-help book called Doing What Matters in Times of Stress: An Illustrated Guide. SH + is delivered by two trained facilitators.

SH + was developed based on Acceptance Commitment Therapy (ACT). It consists of five core skills described in the book Doing What Matters (DWM) in times of stress: (1) grounding (bringing our attention to the present moment); (2) unhooking (unhooking from difficult thoughts and feelings); (3) acting on your values (identifying personal values and acting based on those values); (4) being kind (being kind to oneself and others); and (5) making room (making a room for difficult thoughts and feeling, instead of fighting with them). The participants are not expected to share much personal information with each other or with the facilitator. They focus on learning self-help skill to manage stress [20].

The audio material contains key information on stress management and guides participants through individual exercises and small group discussions [52]. The self-help book entitled *Doing What Matters in Times of Stress: An illustrated Guide* [21] reviews all essential content and concepts. A copy of the book will be provided for each participant. The SH + facilitators will guide participants during each group session by playing the audio, reviewing the skills introduced by the audio, providing culturally relevant examples and clarifications, and reading out discussion questions to make the groups interactive [20].

Before being tested, the SH + intervention underwent adaptation to ensure its acceptability and relevance to the context, as recommended by the WHO. This process followed the WHO guidelines for adapting interventions [53]. This adaptation process included a rapid qualitative assessment, for which we interviewed students, stakeholders, and mental health professionals within the university. The adaptation consisted of the language, visuals, examples, and metaphors used in the illustrated book, audio, and facilitator manual. Further details about the adaptation process will be provided in a separate paper.

#### Enhanced care as usual (ECAU)

Enhanced care as usual (ECAU) consists of an e-leaflet containing information about support services at the university. This includes options for individual counselling and peer counselling and instructions on how to schedule an appointment. Other information includes mental health services outside universities and emergency contact for mental health issues.

#### Contamination

To minimize the risk of contamination, we implemented several measures: During the first session, participants in the SH + group are explicitly asked not to share or discuss the intervention materials with others outside the SH + group. We do not explicitly restrict the control group from independently accessing the SH + materials on the WHO website. Therefore, to know if they were accessing the material, during the T2 and T3 we ask them about whether they knew about the SH+ (see other measures above).

#### Facilitators

SH + will be delivered by two facilitators (a pair), and each university will have two to four facilitators. SH + facilitators have at least a bachelor’s degree in psychology. Individuals with a bachelor’s degree in psychology and who are currently in training to become a psychologist can be a facilitator.

There will be open recruitment to become a facilitator, and recruitment will start during the preparation of the study and before the recruitment of the study participants. The facilitator will receive up to 40 h of training based on the SH + training manual [54], and the total number of hours will depend on the skills and experience of the facilitators already has. The training will be conducted by the coordinator of this study (DA) or a clinical psychologist who has been trained by the coordinator of this study. The training will consist of basic helping skills, SH + facilitating skills, and managing participants’ distress, explaining the aims and background of the SH + intervention.

In addition to delivering SH+, the facilitator will be the one to contact participants to inform them of their randomization allocation. The facilitator will deliver the ECAU (i.e., leaflet) to all participants via email or media communication of the participant’s choice.

The clinical supervisor and facilitator will receive remote support and supervision from the research coordinator as needed. The facilitator will have an online group meeting with the supervisor who is a clinical psychologist to discuss protocol adherence, review barriers or potential barriers, facilitator concerns, any adverse effects and serious adverse events.

#### Treatment fidelity

Treatment fidelity will be assessed in various ways. First, after finishing each session, the facilitator will complete the SH + post-session review form [20] and a fidelity checklist form, which consists of a list of the main activities of each SH + session. Second, the facilitator will record the session of SH+, and the recording will be assessed with the fidelity checklist by the research coordinator.

### Analysis

#### Sample size

By utilizing G*Power [55], considering psychological distress as the primary outcome with an effect size of 0.3, a power calculation for a repeated measures design suggests a minimum sample size of *N* = 148 (power = 0.95, α = 0.05, rho = 0.9) to detect the expected effect size. Accounting for a 50% attrition rate observed during our pilot, a total of 296 participants will be recruited (148 participants in each arm). At each university, there is an intervention group and a control group. Each group will consist of 20–30 participants.

#### Data analysis

Data will be analysed once all data has been collected; no interim analyses will be performed. However, participants can withdraw from the study or the session anytime without giving a reason.

**Quantitative data (RCT).** An intention-to-treat (ITT) approach will be used to conduct data analysis both in primary and secondary outcomes, and the unit of the analysis is individual. The primary analysis will simultaneously assess the treatment effect on the mean PHQ-ADS total score at each time point. All participants randomized to either group will be analysed, regardless of whether they complete the intervention. The ITT population will consist of all participants randomized to the competing intervention strategies who have at least baseline assessment data available. The main conclusion of the trial will be based on the ITT analysis of the primary outcome (i.e., the effect on the PHQ-ADS score at the three-month follow-up).

Adherence will be defined based on attendance at least three sessions of SH+. A per protocol (PP) approach will be used to test the robustness of the intervention results for all outcomes, which will include only SH+/ECAU participants who attend at least three sessions of the SH + intervention [24]. The attendance of the participants will be recorded by the facilitator(s) of the SH+.

Linear mixed models will be used for the PHQ-ADS (primary outcome) and secondary outcomes to evaluate the treatment effect on all endpoints at t2, t3 and t4, with time as a fixed effect, baseline PHQ-ADS score as a covariate and participants as random effects. A t-test will be used to analyse the differences between the two study arms. Multiple imputation will be used to handle the missing values at 3 months. A sensitivity analysis will be conducted to compare the full sample with the sample that excludes ECAU participants who indicated being exposed to SH + materials.

A health economic analysis will be conducted to assess the cost-effectiveness of SH+/ECAU compared with ECAU only, from both a health system and a societal perspective. The results of this analysis will be expressed as incremental cost-effectiveness ratios (ICERs) per quality-adjusted life years (QALYs) gained and per change in the PHQ-ADS composite score at the *t*_*3*_ follow-up assessment point (3 months). QALYs will be estimated using the EQ-5D-5 L questionnaire adapted to the Indonesian value set [46]. A deterministic sensitivity analysis will be performed to identify the most sensitive parameters affecting the ICER. The cost-effectiveness thresholds will follow the current guidelines for Indonesia, which is 1–3 times GDP per capita. The difference and QALY difference between the two groups will be estimated using generalized linear regression. The skewed distribution of the cost data was accounted for by estimating robust standard errors using the Huber-White sandwich estimator.

**Qualitative data (process evaluation). The** Interviews and FGDs will be audio recorded and transcribed verbatim. Thematic analysis will be carried out using ATLAS.ti [56].

#### Data management and confidentiality

The data collected by Castor EDC is automatically anonymized, and each participant will be assigned a unique participant number. The recording of the SH + session will be deleted as soon as it is checked with the fidelity checklist. The recording from the evaluation process, which consists of the interviews and the FGDs, will be deleted after the transcription of the recording is complete. The data management plan of this research can be accessed on https://dmponline.dcc.ac.uk/public_plans.

### Trial monitoring and adverse events reporting

(Serious) adverse events ((S)AEs) are defined as any undesirable experience that a participant has during the study, regardless of whether it is related to the study procedure or the SH + program. All SAEs will be recorded in Castor EDC and reported to the Research Ethics Committee of Universitas Padjadjaran. The research team will follow up with all SAEs until they are stabilized or resolved. If necessary, participants will be referred to a general practitioner. Participants may withdraw from the study at any time. No withdrawal criteria are specified.

To support participants who may require additional mental health care, we collaborate with the University Counselling Centre and have established a referral procedure. In this study, the research assistants have been trained to familiar with the mental health facilities available and know how to refer participants to the University Counselling Centre.

This study involves several potential risks, including emotional distress triggered during sessions and, in some cases, suicidal thoughts. To mitigate these risks, we have implemented a clear referral protocol for participants who express suicidal thoughts or severe distress. Facilitators are trained to respond sensitively and involve mental health professionals when necessary. Participants are informed of these procedures during the consent process and reminded that they can withdraw at any time without consequence.

## Discussion

To the best of our knowledge, this is the first study in Indonesia to evaluate the effectiveness and cost-effectiveness of a face-to-face, group-based psychological intervention for psychological distress in an RCT. The previous studies conducted on transdiagnostic interventions in Indonesia were RCTs or feasibility studies of digital psychological interventions, such as internet-based behavioural activation [57], web-based stress intervention [58], and a counselling application [59]. While these studies have provided valuable insights, there is still a lack of knowledge about the implementation and effectiveness of transdiagnostic, face-to-face psychological interventions in Indonesia. Face-to-face communication may hold particular value for mental health interventions, as it fosters direct interpersonal interactions and promotes a sense of connectedness, which was found crucial for mental health [60, 61].

In this APRESIASI project, SH + has not only been adapted to the Bahasa Indonesia and Indonesian culture in general, but it has also been adapted to the context of university students. These adaptations include examples of stressors commonly faced by students, such as academic pressures, financial concerns, and interpersonal conflicts. By tailoring SH + to the unique challenges of this population, the intervention aims to address the heightened prevalence of anxiety and depression among Indonesian university students. This group often faces barriers to accessing mental health care due to stigma [62–64], lack of mental health literacy [63], and lack of accessibility [62, 65]. Previous beneficial outcomes of SH + among refugees and asylum seekers [66], suggest that SH + has the potential to reduce depressive symptoms and self-identified problems, and improve well-being among university students. Following the evaluation of the program, it is expected that SH + can be embedded into university mental health programs across Indonesia.

As common in psychological intervention trials, participants in this study could not be concealed from the treatment allocation for ethical and practical reasons. This may introduce some bias in self-reported outcomes, e.g. the intervention group may report greater improvements due to positive expectations, while the control group may experience disappointment. However, both groups were informed about the intervention and control condition and had access to university mental health facilities. These measures mitigated expectancy effects and reduced potential biases.

In conclusion, this study contributes to the growing body of research on culturally adapted psychological interventions for young adults in low- and middle-income countries. It highlights the importance of tailoring evidence-based interventions to local cultural contexts and specific populations. Future research could explore the potential for hybrid or digital adaptations of SH + to further expand accessibility and evaluate its applicability to other groups facing psychological distress in Indonesia and beyond. By addressing the mental health needs of university students, the APRESIASI project lays a foundation for promoting well-being and academic success across the nation.

## Electronic supplementary material

Below is the link to the electronic supplementary material.

Supplementary Material 1

Supplementary Material 2

Supplementary Material 3

## Data Availability

No datasets were generated or analysed during the current study.

## Abbreviations

ACT: Acceptance commitment therapy
CONSORT: Consolidated standard of reporting trials
CSRI: Client service receipt inventory
DWM: Doing what matters in times of stress
(S)AEs: Serious adverse events
ECAU: Enhanced care as usual
EDC: Electronic data capture
EQ-5D-5L: EuroQol 5-dimensional descriptive system—5-level version
GAD-7: Generalized Anxiety Disorder, seven-item measurement instrument
ICF: Informed consent form
ITT: Intention-to-treat
MIMIS: Mainz Inventory of Microstressors
PHQ-9: Patient Health Questionnaire depression module, 9-item measurement instrument
PP: Per protocol
SH+: Self-Help Plus
SPIRIT: Standard protocol items: recommendations for interventional trials
Unpad: Universitas Padjadjaran
WHO: World Health Organization
WHODAS 2.0: WHO Disability Assessment Schedule 2.0
