# Supplementary material for: Effectiveness of adapted self-help plus (SH+) to reduce psychological distress among university students in Indonesia (APRESIASI): protocol of a randomized controlled trial
Source: BMC Psychol. 2025 Jul 8;13:752. doi: 10.1186/s40359-025-03026-y (PMC12236022; doi:10.1186/s40359-025-03026-y)
Supplement: Supplementary file 1 — Supplementary Material 1 [file 40359_2025_3026_MOESM1_ESM.pdf]

Privacy statement: APRESIASI

Date: 19 March 2024

The Vrije Universiteit Amsterdam (hereinafter: "**VU"**), Universitas Padjadjaran (**UNPAD**), and Bandung Institute of Technology (**ITB**) attaches great importance to the protection of your privacy and the security of your personal data. In this privacy statement we describe how we handle your personal data [describe the project/process]. We process your personal data in accordance with applicable privacy legislation, including the General Data Protection Act (hereinafter: "**GDPR "**)and the General Data Protection Implementation Act, and Indonesian Psychology Code of Ethics

1. Who is responsible for the processing of my personal data?

Stichting VU is responsible for the data processing operations described in this privacy statement. Stichting VU maintains the Vrije Universiteit Amsterdam as a privately run university in accordance with the Higher Education and Research Act of The Netherlands (*'Wet op het hoger onderwijs en wetenschappelijk onderzoek').* Stichting VU has its registered office at De Boelelaan 1105 in (1081 HV) Amsterdam and is registered with the Chamber of Commerce under number 53815211.
Universitas Padjadjaran (UNPAD) has its registered office at Jalan Ir. Soekarno Km. 21 Jatinangor, West Java 45363, Indonesia.
Bandung Institute of Technology (ITB) has its registered office Jl. Tamansari 64 Bandung 40132, West Java, Indonesia.

2. What (categories of) personal data will be processed?

We will process the following personal data:

a. Name

b. Contact details: telephone number and email address

c. Demographic data:
   - gender/sex,
   - age,
   - religion,
   - ethnic group,
   - area of origin,
   - faculty & field of study,
   - GPA,
   - living allowance.
   - Living condition (type of housing, with whom)
   - Comorbid condition (diabetes, asthma, low/ high blood pressure, hearth condition, autoimmune, ect)

d. Screening data:
   - Psychological distress
   - information about the imminent risk of suicide or self-harm or other life-threatening risk based on interview

- Information about cognitive and neurological functioning using observation checklist.
- Information about using prescribed or over-the-counter medication for mental health problem
- Information about using specialised psychological treatment Mental health status data
- Mental health status: symptoms of psychological distress, symptoms of depression, symptoms of anxiety, perceived stress, general functioning, micro stressors, resilience, self-identifying problems, and quality of life.

e. Cost-effectiveness

f. Recording during intervention to check for fidelity of SH+ intervention delivery. After the fidelity check, the audio recording will be deleted.

g. Transcript interview of the evaluation of the interview

3. For which purposes are my personal data processed, and on the basis of which legal grounds?

The personal data will only be used for the following purposes:

a. Name and contact details: The telephone number will be used to invite and remind the participant about the intervention session as well as remind to fulfill the questionnaire. The email address and will be used to send the link to the questionnaire and to send the reminder to complete the questionnaire.

b. The screening data will be used to determine whether you are eligible to participate in the study

c. The demographic data, mental health status, and cost-effectiveness data will be analysed to answer the research question and hypotheses;

d. The recording during the intervention will be used to check the fidelity of facilitators;

e. Transcript interview of the evaluation of the interview will be used to evaluate the intervention and the study.

We process your personal data on the basis of the following legal grounds:

- You have given consent to the processing of your personal data. In that case you have the right to withdraw your consent at any time by contacting us via the contact details below.

4. Who has access to my personal data?

The personal data will only be accessed by employees of the VU, UNPAD, and ITB who by reason of their function has a role in the processing of your personal data for the above-mentioned purposes and for whom it is necessary that they have access to the personal data.

5. Will my personal data be shared with third parties?

When processing your personal data, we may use service providers (processors) who process your personal data on behalf of and under the responsibility of the VU. The VU concludes processing agreements with these service providers to ensure that your personal data is processed carefully, securely and in accordance with the General Data Protection Regulation (GDPR). We remain solely responsible for these processing activities.

Your personal data will not be shared with other parties. Should this be the case at any time, we will inform you of this. We only disclose personal data to third parties to the extent permitted by privacy law, for example because you have given your prior consent, or we are legally obliged to do so.

6. Will my personal data be transferred to countries outside the European Economic Area?

Yes, your personal data will be transferred to UNPAD. The cost-effectiveness data will be analysed by the expert in UNPAD. All the personal identification data will be deleted and pseudonymization will be done before data transfer.

7. For how long will my personal data be retained?

We will not retain your personal data for longer than is necessary to achieve the predetermined purposes or as long as required by law.

Categories of personal data	Retention period
Name and contact detail	After the last follow-up: 6 months after the end of intervention
Screening data	15 years after the study
Demographic data	15 years after the study
Mental health status	15 years after the study
Recording during intervention	After the check for fidelity
Recording for evaluation of intervention	After the transcribing and check for the validity of transcribing
Transcript of interview	15 years after the study

8. How will my personal data be secured?

The VU takes appropriate technical and organizational measures to protect your personal data against loss and any form of unlawful processing.

1. Raw data will be saved in the Qualtrics and Castor server
2. Data will be saved in Research Drive and OneDrive and confidentiality data will be password protected
3. The data transfer will use file encryption.

For the safety of the data, there multifactor authentication to log in the application and confidentiality agreement for the all personnel who involved in this study.

9. Who can I contact with questions about the processing of my personal data?

You can ask questions about how we process your personal data via d.andriani@vu.nl or dhini.andriani@unpad.ac.id or +31687764395.

10. How can I exercise my privacy rights?

On the basis of the GDPR you have the right – under certain conditions – to access your personal data that we process, to correct your personal data if it contains factual inaccuracies, to delete your personal data, to limit the processing of your personal data, to portability of your personal data and to object to the processing of your personal data.

If you wish to exercise any of these privacy rights, you can contact the Data Protection Officer of VU Amsterdam via:

Data Protection Officer
De Boelelaan 1105
1081 HV AMSTERDAM
functionarisgegevensbescherming@vu.nl

***
