# Supplementary material for: Effectiveness of adapted self-help plus (SH+) to reduce psychological distress among university students in Indonesia (APRESIASI): protocol of a randomized controlled trial
Source: BMC Psychol. 2025 Jul 8;13:752. doi: 10.1186/s40359-025-03026-y (PMC12236022; doi:10.1186/s40359-025-03026-y)
Supplement: Supplementary file 2 — Supplementary Material 2 [file 40359_2025_3026_MOESM2_ESM.pdf]

INFORMATION ABOUT APRESIASI PROJECT

English title:

Effectiveness of Adapted Self-Help Plus (SH+) to Reduce Psychological Distress among University Students In Indonesia

Dear student,

The research team consists of Dhini Andriani (Vrije Universiteit Amsterdam and Padjadjaran University), Fredrick D. Purba (Padjadjaran University), Marit Sijbrandij (Vrije Universiteit Amsterdam) and Anke Witteveen (Vrije Universiteit Amsterdam).

With this information letter, we would like to ask you if you would like to participate in scientific research. We ask you to participate in a study on the effectiveness of Self-Help Plus (SH+), a group-based intervention for psychological symptoms of distress such as depression, stress, and anxiety.

Participation is voluntary. To participate, your written permission is required.

Before you decide if you want to participate in this research, you will receive an explanation of what the research entails. In this letter, you can read what kind of research it is, what it means for you, and what the advantages and disadvantages are of participating in this research. It is a lot of information, but we ask you to please read this information carefully and then decide if you want to participate.

If you need any clarification, please ask the researcher for an explanation. You can also talk about it with others, such as your friends, lecturer, or family.

If you would like to participate, you can complete the consent form at the link sent with this information letter.

Ask your questions

You can make your decision based on the information you find in this information letter. In addition, we recommend that you do this:

- Ask questions to the researcher who provided you this information *[and who will contact you next week]*.

- Talk to your friends, lecturer, or family about this research.

1. **General information:**

   The Vrije Universiteit (VU) Amsterdam in cooperates with Universitas Padjadjaran (UNPAD) have set up this research. Investigators are conducting the study. This research requires 296 research participants. The Research Ethic Committee of Universitas Padjadjaran has approved this research.

2. **Purposes and background of the study**

   This study will examine whether the SH+ intervention, combined with enhance care as usual (ECAU) reduces psychological symptoms of distress such as depression and anxiety, and increases well-being.

3. **What is the background of the investigation?**

   Depression, stress, and anxiety are common psychological symptoms of distress among university students in Indonesia. These symptoms may be caused, for example, by academic difficulties, conflicts with friends, difficulties managing time, or difficulties adjusting to university life. Psychological distress can cause a lot of suffering and lead to obstacles in carrying out daily activities.

   Previous research indicates that the programs we will investigate within APRESIASI are effective in reducing mental health problems. With this research, we want to find out whether the SH+ program is also a useful tool for university students in Indonesia.

4. **How's the investigation Going?**

   How long does the investigation take?

   Are you participating in the investigation? Then that takes about 8 months in total.

   Step 1: Are you eligible to participate?

   First, we want to know if you are eligible to participate. That is why the researcher first asks you questions about symptoms of distress. We will set up a meeting for this. This will take about 15–20 minutes. If it turns out that you are not eligible to participate, the researcher will tell you more about this.

   Step 2: SH+ program

   The SH+ program is a group-based management stress which consists of 20–30 participants. The group will be conducted by two trained facilitators. The SH+ program has

five (5) sessions that will be held once a week that last for 90–100 minutes. You will not talk much about your personal problems with other participants nor with the facilitators, you will be focused on learning about management of stress. SH+ has two components, which are a book title *Doing What Matters in time of Stress: an illustrated guide* and audio instruction. You will have the book that you can bring and read at home. During the SH+ program, you will listen to audio instruction and follow the lead of the facilitator. From the audio instruction and facilitator you will receive information about stress, such as the symptoms, the cause, and exercise to manage it. In the SH+ program, both book and audio instruction based on acceptance commitment therapy (ACT). ACT has been proven effective in reducing psychological distress, such as symptoms of depression and anxiety. For this research, we create 2 groups:

- **Group 1**. The people in this group will participate in a 5-week group management stress intervention. In addition, they receive enhance care as usual (ECAU) and can make use of the usual care.
- **Group 2**. The people in this group do not participate in 5-week group management stress intervention. However, they do receive enhance care as usual (ECAU) and can utilize the usual care.

Enhance care as usual (ECAU) is information about mental health, where and how to get help inside and outside the university. In this study, the ECAU was provided via email in the form of an e-leaflet. All participants in this study received the ECAU.

If you qualify, and then the computer will decide which group you will be in. This is a random process: You have a 50% chance of being in group 1 and a 50% chance of being in group 2. The researcher does not know whether you will be in group 1 or group 2. This is necessary to prevent this knowledge from influencing the results of the study. Therefore, we ask that you do not tell the researcher which group you are in.

Step 3: Examination and measurement

Questionnaires: The study requires you to complete several online questionnaires, 4 times over 8 months. Completing these questionnaires takes 20–30 minutes at a time. The questionnaires are about symptoms of distress, such as anxiety, low mood and stress, your general quality of life and use of healthcare.

5. What agreement do we make with you?

We'd like the investigation to go well. That is why we make the following agreements with you:

- You attend each session, which is every Saturday for five weeks. .
- You fill in all (online) questionnaires.
- You contact the researcher in these situations:
  - o You will be admitted to or treated in a hospital.
  - o You suddenly have problems with your health.
  - o You would rather not be part of the investigation anymore.
  - o Your phone number, address, or e-mail address changes.

6. What are the advantages and disadvantages when you participate in the research?

Participating in the study can have advantages and disadvantages. We list them below. Think about this carefully, and you can also talk about it with others. SH+ may reduce symptoms of distress, such as anxiety, low mood and stress, but this is not certain. If you enter the control group (Group 2), you yourself will not benefit from participating in this study. However, with your participation, you help the researchers to gain more insight into whether short-term mental health programs reduce symptoms of distress and improve wellbeing and resilience among university students in Indonesia.

Taking part in the study can have disadvantages:

- Participating in the study costs you (extra) time.
- Completing questionnaires can cause temporary tension or be confrontational. You can always skip questions if you prefer.
- You may temporarily experience more tension or anxiety while going through the programs. The facilitators for the APRESIASI project are trained to deal with feelings of tension and anxiety. There is also always an experienced employee available who can provide psychosocial support or can refer you to more specialized care if you wish.
- You must adhere to the agreements that are part of the research.

Do you not want to participate?

You decide if you participate in the study. Do you not want to participate? The researcher can tell you more about treatment options available, such as psychological support, and their advantages and disadvantages.

7. When will the research end?

There might be situations in which the researcher will inform you that research ends for you. In these situations, the research will end for you:

- When we ask questions to check if you are eligible to participate (as explained under section 4, Step 1), you indicate that you do not experience much psychological distress, such as depression or anxiety, or daily limitations;
- The 4 appointments for completing the online questionnaires are completed;
- You want to stop the research yourself. That is allowed at any time. Report this immediately to the researcher. You do not have to tell us why you are stopping;
- The investigator thinks it is better for you to stop. The researcher will still invite you for a meeting to discuss this;
- One of the following authorities decides to stop the investigation:
    - the sponsor
    - the government, or
    - the ethics committee that assesses the research.

What happens if the research ends for you?

The researchers use the data that has been collected up to the moment of stopping. The entire study is over when all participants have completed the study.

8. What happens after the research?

About 2 years after your participation, the researcher will inform you by e-mail about the main results of the study.

If you need more psychological help after this intervention has ended for you, we will refer you to your psychologist at university. In case you do not agree, the research team will make you get an appointment with other psychologists in another institution.

9. What do we do with your data?

Are you participating in the study? Then you also give permission to collect, use and store your information.

What information do we store?

We store this information:

- your name
- your gender
- your telephone number
- your e-mail
- information about your health

Why do we collect, use and store your information?

We collect, use and store your information to answer the questions of this research and to publish the results. The facilitator can also watch you during the SH+ intervention and write her observation in the form. This way, the facilitator can help you if you require more support. Furthermore, we want to make an audio record of the SH+ intervention. This way, we can check if the facilitator performs the SH+ the way that they are supposed to (fidelity checks).

How do we protect your privacy?

To protect your privacy, we provide your information, including audio records, to a code. We keep the key to the code in a secure place at the research institution. When we process your data, we always only use that code. Furthermore, in reports and publications about the research, no one can recall that it was about you.

When do we disclose your data?

In case the research team or the SH+ helpers find that there is a potential risk of a serious adverse event, self-harm or suicide, the research coordinator will refer you, with your permission, to the University Counselling Service at your university. The University Counselling Service's may assess you for mental health problems, and provide counselling or psychotherapy for your condition if indicated.

How long do we store your information?

We store your data for 15 years at the location research centre. The audio record of SH+ sessions will be stored until the end of the research, and we have performed all fidelity checks. After this, audio records will be destroyed.

10. Compensation

Participation in the APRESIASI project is free of charge. To participate in this study, you will receive a voucher or e-money of IDR 50,000 for completing each of the four (online) questionnaires. You will receive a total of voucher or e-money of IDR 200,000. In addition, during the session of SH+ snacks and tea/ coffee will be provided. Will you stop before the research is completed? Then you will receive a compensation only for the questionnaires you completed.

11. Insurance

You are not additionally insured for this study. Because taking part in the study has no additional risks.

12. Do you have any question?

You can ask questions about the research to the VU and UNPAD research team.

13. How do you give permission for the research?

First, you can think carefully about this research (at least one week). Thereafter, you tell the researcher if you understand the information and if you want to participate or not. Would you like to participate? You can complete a digital version of the consent form attached with this information letter in your email. Both you and the researcher will receive a signed version of this consent form.

Thank you for your time.

14. Appendices to this information letter

A. Contact information

Research coordinator

Ms. Dhini Andriani d.andriani@vu.nl, (phone number)

Main researchers:

Mr. Fredrick D Purba, M.Psi., Ph.D., fredrick.d.purba@unpad.ac.id

Ms. Dr. Anke Witteveen, a.b.witteveen@vu.nl

Ms. Prof. Dr. Marit Sijbrandij, e.m.sijbrandij@vu.nl

B. Consent form

APRESIASI: Effectiveness of Adapted Self-Help Plus (SH+) to Reduce Psychological Distress, Improve Functioning, and Quality of Life among University Students In Indonesia

- I have read the information letter. I could also ask questions. My questions have been sufficiently answered.
- I have read the privacy statement. I understand how data protection and data sharing in this research.
- I had enough time to decide whether to participate.
- I know that participation is voluntary. I also know that I can decide at any time not to participate or to discontinue the study. I do not have to give a reason for that.
- I know that I can be referred to a psychologist or counsellor if I need additional psychological support.
- I knew that during the sessions of the stress management course, the sessions would be recorded to check the fidelity of the intervention delivery.
- I know that some people can view my information. Those people are listed in this information letter.
- I give permission for the collection and use of my information in the way and for the purposes stated in the information letter (see also section 4):

Transfer data: 1) My data will be shared with the APRESIASI project partners, namely Vrije Universiteit Amsterdam, Padjadjaran University and Bandung Institute of Technology, in connection with this research. I have been informed that my data is subject to Dutch data protection levels. The data will be shared in encrypted form, without my name or any other personal information that would allow me to be directly identified.

- Consent
- Not consent

Follow-up. 2) Permission to approach me again after this research for a follow-up research.

- Consent
- Not consent

Data 15y. 3) Permission for my data to be kept at the research site for 15 years after this research so that it can be used for future scientific research on the mental health of university students.

- Consent
- No consent

Data disclosing. 4) Permission to refer me to the University Counselling Service if I am known to be at risk of harm to myself (You can still participate in the study even if you do not agree to this section)

- o Consent
- o No consent

I want to participate in this research.

Name participant: ___________________________

Signature participant: ___________________________

Date: ___ / ___ / ______ (DD/MM/YYYY)

I declare that I have fully informed this participant about the mentioned research.
If information becomes known during the study that could influence the participant's consent, I will inform him / her in good time.

Name researcher (or its representative): _________________________

Signature researcher (or its representative): _________________________

Date: ___ / ___ / ______ (DD/MM/YYYY)

The participant will receive a complete information letter, together with a copy of the signed consent form
